# The Impact of Health Risk Perception on Blockchain Traceable Fresh Fruits Purchase Intention in China

**DOI:** 10.3390/ijerph19137917

**Published:** 2022-06-28

**Authors:** Qianqian Zhai, Ali Sher, Qian Li

**Affiliations:** 1 College of Economics and Management, Nanjing Agricultural University, Nanjing 210095, China; 2017206023@stu.njau.edu.cn; 2 Business School, Nanjing University of Information Science & Technology, Nanjing 210044, China; s.ali@nuist.edu.cn; 3 College of Economics, Beijing Technology and Business University, Beijing 100048, China

**Keywords:** health risk perception, blockchain, traceable, fresh fruit, income

## Abstract

This paper systematically investigates the impact of consumers’ health risk perceptions on the purchase intention of blockchain traceable fresh fruits in China. It uses online-survey data collected from four pilot cities that are part of the food traceability system in China. The ordinary least squares (OLS) and the ordered probit model was applied to examine the posited relationships. The results show that consumers’ health risk perception has a significant positive effect on the purchase intention of blockchain traceable fresh fruits. The stronger consumers’ health risk perception, the stronger their purchase intention of blockchain traceable fresh fruits. Likewise, heterogeneity exists among gender, age, income, and education in their corresponding effect of consumers’ health risk perception on blockchain traceable fresh fruit purchase intention. This suggests that male, high-aged, high-income and high-educated groups have a higher health risk perception, and therefore a higher purchase perception for blockchain traceable fresh fruits than female, low-aged, low-income and low-educated, respectively. Furthermore, family structure, consumers’ traceability cognition and purchase experience of traceable products affect the purchase intention of blockchain traceable fresh fruits. The study has several insights on the broader promotion, acceptance and development of the food traceability system and provides practical cues for policy and practice.

## 1. Introduction

Increased urbanization and the rapid growth of the Chinese middle-income group have unprecedentedly changed their purchasing attention, particularly in food quality, nutrition, and food safety [1,2,3]. Today, consumers are fully aware of the effects of excessive pesticides and chemical fertilizer use on food crops [4]. In particular, the frequent food quality and safety incidents have raised the perception of their health risks and adversely affected their buying behavior towards traditional marketing channels [5]. Since health risk perception significantly affects consumers’ health behavior [6,7,8], the use of blockchain traceability is becoming more popular because it can build consumer trust by providing detailed information on the product quality and safety attributes [9]. While blockchain traceability significantly overcomes consumer concerns, knowledge gaps remain as to whether and how this process accurately unfolds for fresh fruit purchase intentions.

Traceability plays an important role in the food supply chain [10,11,12]. The traceability system can reveal more information on product quality and safety; it incurs low cost, and it can help increase consumers′ product confidence [10]. The traceability system has been widely used in pork, fish, and other agricultural products and achieved good results. However, there are also some problems in the traditional traceability system. At the supply level (farm-gate), the traceability system is too dependent on the centralized system, the data is easy to be tampered with, the information can be forged, and there is formation of information islands among the participants [13,14]. From the perspective of demand, consumers have low awareness of and attention to the traceability system, increasing their doubts about the authenticity of agricultural products with traceability labels and leading to low purchase intention [15,16]. However, studying the fresh fruits under the traceability sphere presents a unique case, as it incurs a few stakeholders from farm to consumer and have frequent interaction in daily life.

Blockchain technology generates, integrates and innovates information using cloud computing, big data, the Internet of Things (IOT), information technologies, and its application scenario has been extended to the field of agricultural food traceability [17,18]. Blockchain traceability has security features such as distributed data storage, consensus mechanisms, data invariance, is tamper-proof and has encrypted timestamps [13,19], which can provide more secure, transparent and accurate information [20,21] and ensure the authenticity and credibility of food characteristic information [22]. Through various smartphone applications, consumers can scan the blockchain traceability quick response code (QR code) on the package to obtain more information about food [19,23]. Doing so reduces consumers’ health risk perception and enhances consumers’ confidence in purchasing and consuming products [24,25]. However, as cutting-edge technology, blockchain traceability is still in its infancy and is at the stage of small-scale pilot and exploration at the enterprise level [26,27].

A scalable promotion of the blockchain traceability system can be realized by understanding consumers’ purchase intention of blockchain traceability products, especially fruit products that people consume daily. Recent statistics indicate that the per capita consumption of fresh melons and fruits among urban residents in 2020 increased by 26% compared with that in 2013, and was higher than the increase in meat, eggs, and milk [28]. However, the China food safety development report 2018 and the big data on food safety incidents point out that food safety incidents of edible agricultural products are much higher than that of the other five categories of foods. Inter alia, the fresh fruits and fruit products had the largest number of incidents [29]. Recent studies explored the blockchain traceability for pork in China and the USA [9], as well as seafood [30], organic food products [31], organic rice [32], and beef [33]. However, there remains scant research on the purchase intention of traceable fresh fruits. The detection rate of pesticides in fresh fruits is relatively high [34], and the problem associated with fresh fruit quality and safety is more prominent. Therefore, it is more practical to take fresh fruits as the research case to explore the impact of consumers’ health risk perception and intention to purchase blockchain traceable fresh fruits.

Based on the micro survey data of fresh fruit consumption in China, this paper aims to examine the impact of health risk perception on blockchain traceable fresh fruits purchase intention. The rest of the paper is arranged as follows. The next section consists of a literature review. Section 3 describes the data and methods. The results are discussed in Section 4. Lastly, Section 5 presents the conclusions and policy implications.

## 2. Literature Review

Prior literature has explored that health risk perception is usually related to tobacco [35], illness [36], and extreme weather [37]. It plays an important role in motivating people to adopt healthy behaviors [6]. But previous studies paid more attention to the antecedents of health risk perception than the outcomes. In terms of the antecedents of health risk perception, gender, age, education level, and unit qualification cause differences in individual health risk perception level [38], especially gender, which has gained more attention [39,40,41]. Godovykh et al. [42] classified the main factors of health risk perception into cognitive, affective, individual, and contextual components. In terms of outcomes of health risk perception, it was revealed that risk perceptions are directly associated with physical activity participation [39], mental health [43], behavioral changes [44], risk-protective motivation or behavior [45,46]. A few studies explored the impact of health risk perception on the consumption of alcohol [47], fish [48], and sugar [49]. However, few studies focused on traceable products, especially blockchain traceable products.

The research on the combination of blockchain and traceability gradually appeared in 2019. Most of the research focused on the agricultural food sector (such as vegetables, corn, rice, grapes, bananas, cocoa chocolate, tea, and pasta), followed by the general agricultural product supply chain and animal husbandry sector (chicken and pork) [50]. Scholars unanimously affirmed the positive role of blockchain in the traceability of agricultural products [19,24]. On the one hand, from the theoretical level, recent studies proposed schemes for applying blockchain in agricultural food traceability [51,52]. Some researchers simulated the possible application scenarios of blockchain in food traceability [53]. However, there is a lack of evidence to compare the differences in adoption mechanisms among various stakeholders, which hinders the implementation of blockchain in the agricultural supply. Lin et al. [14] proposed a food safety traceability system based on blockchain by developing a prototype system and a data management architecture based on the supply chain. The problem of data explosion in the IOT blockchain can be alleviated through the traceability system.

Other scholars took olive oil [54], beef [55], coffee [4], and other products as examples to explore consumers’ willingness to pay for blockchain traceable products. For example, Williamson [56] studied the willingness of Chinese consumers to pay for mutton certified by the blockchain traceability of the complete supply chain process information from an Australian farm to a restaurant in Shanghai. The results showed that 51.4% of consumers were willing to pay a premium for blockchain certified products, of which 68.4% were willing to pay an additional 5–15% for blockchain certified products. Lin et al. [31] studied the factors influencing Chinese consumers’ willingness to use the organic food blockchain traceability system based on the information system success model and the theory of planned behavior. The results showed that attitude and perceived behavioral control, system quality, information quality and service quality significantly (positively) influenced consumers’ trust, which in turn affected their willingness to use traceability.

Compared with existing studies, the paper contributes to the literature on marketing and blockchain traceability in several ways. First, blockchain traceability belongs to the technical frontier of a traceability system, and there are relatively few research studies with regard to it. To the best of the authors’ knowledge, this is the first study exploring consumers’ purchase intention of blockchain traceable fresh fruit. Second, the research data comes from traceable pilot cities, which are representative and effective for a broader generalization and policymaking. Third, considering the heterogeneity in gender, age, income and education, and studying the impact of health risk perception on blockchain traceable fresh fruit purchase intention, this research is more inclusive and provides more practical insights for policy and practice.

## 3. Data and Methods

### 3.1. Data Collection

This study was conducted during COVID-19, and the data set was collected through a comprehensive online survey. Before the formal survey, the research team conducted an offline pre-investigation in Nanjing in April 2021, randomly intercepting respondents in three representative places by visiting supermarkets, communities and shopping malls. To ensure the representativeness of sampling, from 10 a.m. to 8 p.m., the investigator(s) randomly selected one consumer from every three who came into sight as the potential sample. Those who were willing to participate in the survey and complete the survey were taken as valid respondents. Finally, 33 valid samples were obtained. Based on the pretesting information, the survey questionnaire was revised to make it more inclusive and insightful, and the formal online survey was implemented. In the final survey, we considered factors such as regional economic development and residents’ consumption habits to include respondents from four pilot cities, including Nanjing, Beijing, Xian, and Fuzhou. These four cities are part of traceability systems pilot cities implemented in P. R. China. According to China Statistical Yearbook 2020 [57], the per capita disposable income of urban residents in Beijing, Nanjing, Fuzhou and Xi’an is $10,996, $7614, $6794 and $4962, respectively. It represents the regions with different levels of economic development, which enhance the data credibility and results produced. Furthermore, we consider the harvesting time of fruits and thus list one after another in summer. The final online survey was conducted in April and May of 2021. The official online survey was conducted by Questionnaire Star. To participate in the online survey, respondents had to meet three pre-conditions: (1) they had to be the main household food buyers, (2) they had to buy fresh fruit in the past month, and (3) they were required to be over 18 years old. To improve the quality of the online survey data, trap questions and the shortest time limit were included in the questionnaire. A total of 1126 samples were obtained. According to the screening rules set by the questionnaires, we eliminated wrong or unreasonable questionnaires (not fully completed), and therefore, we finally obtained 1058 valid questionnaires, with an effective rate of 94.0%, including 284, 257, 261 and 256 from Nanjing, Beijing, Xian and Fuzhou, respectively.

### 3.2. Data Description and Summary Statistics

**Figure 1:**
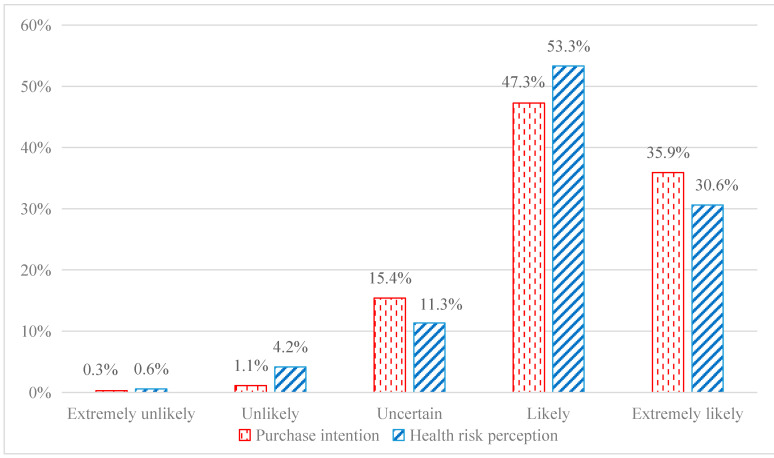
Purchase intention and health risk perception of consumers.

The questionnaire measures dependent variables as consumers’ purchase intention of blockchain traceable fresh fruits by asking “how likely are you to purchase blockchain traceable fresh fruits”. Responses were taken using a 5-point Likert-type scale ranging from 1 (extremely unlikely) to 5 (extremely likely). As shown in Figure 1, almost half of (47.3%) respondents replied “likely” and 35.9% responded “extremely likely”. In total, a majority (83.2%) of the respondents were willing to purchase blockchain traceable fruits. Related studies show that a sizeable market segment is willing to pay a premium price for blockchain traceable products [4,9]. These studies also confirm the enthusiasm of consumers towards blockchain traceable products.

The questionnaire measures consumers’ health risk perception as independent variables by asking “at present, incidents endanger food safety such as pesticide residues, illegal preservatives, and industrial wax occur occasionally, so I am more worried about these health risks when buying fresh fruits”. Responses were noted using a 5-point Likert-type scale ranging from 1 (extremely disagree) to 5 (extremely agree). As shown in Figure 1, 53.3% and 30.6% of consumers may and are very likely to worry about fresh fruit health risks, respectively, and the average value of consumers’ health risk perception in Table 1 is 4.093. Generally speaking, consumers have a high health risk perception.

Other potential factors affecting consumers’ purchase intentions are introduced, referring to the existing relevant research experience [9,54,60]. Individual characteristic variables, including consumers’ gender, age, education level, occupational type and marriage. Family characteristic variables include family size, number of children, and family income. Individual cognitive experience variables include traceability cognition, traceable product purchase experience, and food poisoning experience. The specific definitions and descriptive statistics of all variables are shown in Table 1.

### 3.3. Model Specification

Since consumers’ purchase intention variables belong to typical sorting data, following the literature [61,62,63], an ordered logit model (hereinafter referred to as Ologit) is constructed. The specific settings of the model are as follows:

(1) $$Buy_{i}^{*}=\alpha_{0}+\varphi_{1}Health_{i}+\sum_{n=1}^{n}\mu_{n}Control_{in}+\varepsilon_{i}$$

In Formula (1), $Buy_{i}^{*}$ represents the latent variable of consumers’ intention to purchase blockchain traceable fresh fruits, and $Health_{i}$ represents consumers’ health risk perception. $Control_{in}$ are *n* control variables that may affect consumers’ purchase intention. $\alpha_{0}$ is the constant term, $\varphi_{1}$ and $\mu_{n}$ are the parameters to be estimated, $\varepsilon_{i}$ is the residual disturbance term, and it is assumed to obey the standard normal distribution:

(2) $$Buy_{i}=\{\begin{array}{c} 1,\;If\;Buy_{i}^{*}\leq C1\\ 2,\;If\;C1<Buy_{i}^{*}\leq C2\\ 3,\;If\;C2<Buy_{i}^{*}\leq C3\\ 4,\;If\;C3<Buy_{i}^{*}\leq C4\\ 5,\;If\;C4<Buy_{i}^{*} \end{array}$$

In Formula (2), $Buy_{i}$ represents the purchase intention of consumers, and *C*1~*C*5 are the cut-off points. When $Buy_{i}^{*}<C1$, consumers are unlikely to purchase blockchain traceable fresh fruit ($Buy_{i}^{*}=1$), and others in the same analogy. From Equation (2), the likelihood function of the sample can be obtained and the MLE estimator can be obtained, which is the Ologit model. Since the economic meaning of the estimated coefficient of the Ologit model is not intuitive, this paper mainly reports the marginal effect of each independent variable on the dependent variable.

## 4. Results and Discussion

### 4.1. Basic Regression Analysis

Considering the high consistency of the estimation results of OLS regression and the ordered selection model [64], this study estimates the OLS model and Ologit model simultaneously to take the OLS estimates as a reference. In the specific estimation of the model, the independent variable health risk perception (columns 1 and 4), control variables (columns 2 and 5) and regional dummy variables (columns 3 and 6) are introduced successively. The estimation results are shown in Table 2. Among the six regression results, the impact of health risk perception on consumers’ intention to purchase blockchain traceable fresh fruits is significantly positive at the statistical level of 1%, indicating that the stronger consumers’ health risk perception is, the stronger consumers’ intention to purchase blockchain traceable fresh fruits is as well. Based on the estimated results in column (6), compared with consumers with low health risk perception, consumers with high health risk perception have a 6.4% higher probability of purchasing blockchain traceable fresh fruits. This finding indirectly supports previous studies that link health risk perception and fish consumption [48]. Health risk perception has the strongest relationship with intentions of safe consumption [49]. For products with health risks, consumers tend to reduce their consumption and turn to products with low health risks, such as traceable products that can reveal product quality information. This finding also supports the conclusion of the majority of empirical studies which is that there are positive associations between health risk perceptions and consumers’ buying behaviors [6,7].

Most of the control variables significantly affect consumers’ purchase intentions, which is consistent with the existing studies [4,9,65]. Amongst the education groups, college-educated and those with a graduate degree or above are more willing to purchase blockchain traceable fresh fruits. From the perspective of marginal effect, the group with a graduate degree or above have the strongest traceable fruit buying intentions. People with significant educations, especially at the postgraduate level, have higher health risk perception [58]. In terms of family demographic structure, compared with families with fewer children, families with two children are more willing to purchase blockchain traceable fresh fruits, and families with more children do not play a significant positive role in the purchase intention of blockchain traceable fresh fruits. Generally speaking, members from families with children present a different health risk perception and are more likely to hear about the food risks [58].

Middle-income and high-income groups are more willing to purchase blockchain traceable fresh fruits among the income groups. The marginal effect reveals that the two groups’ intention to purchase blockchain traceable fresh fruits is similar. The impact of consumers’ traceability cognition on the purchase intention of blockchain traceable fresh fruits is significantly positive at the statistical level of 1%, indicating that the more consumers know about traceability, the higher the probability of purchasing blockchain traceable fresh fruits. Consumers’ purchase experience of traceable products has a significant positive impact on the purchase intention of blockchain traceable fresh fruits. Previous purchase experience enhances consumers’ trust in traceable products and tends to increase their likelihood of purchasing blockchain traceable fresh fruits. This is because past behavior positively influences future purchase intention, such as for traceable products [66]. These findings imply that creating awareness regarding the health risks of fresh fruits by targeting educated and higher-income groups would help with the rapid expansion, promotion, and acceptance of traceable fresh fruits in China.

### 4.2. Heterogeneity Analysis

To further investigate the impact of consumers’ health risk perception on the purchase intention of blockchain traceable fresh fruits among different consumer groups, the samples are divided into four groups: (1) male and female, (2) young and old, (3) low-income and high-income, and (4) the low-educated and high-educated. The age group is bounded by the average age of the whole sample, and those higher than the average are the high-aged, those lower than or equal to the mean value are the low-aged. The income group is bounded at $22,350. Those higher than $22,350 are the high-income, and those lower than or equal to $22,350 are the low-income. The education group is bounded by undergraduate education. Those with undergraduate education or above are the high-educated, and those with undergraduate education or below are the low-educated. The estimated results are shown in Table 3.

Regarding gender grouping, health risk perception is significant and positive for male and female intention to purchase blockchain traceable fresh fruits at 1% and 5%, respectively. The marginal effect shows that males with strong health risk perception have a significantly higher probability of purchasing blockchain traceable fresh fruits by 10.1% than that with weak health risk perception, and females with strong health risk perception have a 4.5% higher probability of purchasing blockchain traceable fresh fruits than that with weak health risk perception. It indicates that males with strong health risk perception have a stronger motivation to purchase blockchain traceable fresh fruits; health risk perception has a greater impact on males’ purchase intentions. The possible explanation is that blockchain traceable products are a new feature that provides sufficient information to help overcome the uncertainty related to food safety, and, therefore, positively attracts males to experience more frequently than females due to their traditional social role and more conforming behavior [67].

In terms of age grouping, health risk perception is significantly positive for the low-aged and high-aged intention to purchase traceable fresh fruits at 5% and 1%, respectively. The marginal effect indicates that the low-aged with strong health risk perception have a 4.8% higher likelihood of purchasing blockchain traceable fresh fruits than those with weak health risk perception. Likewise, the high-aged with strong health risk perception have an 8.0% higher likelihood of purchasing blockchain traceable fresh fruits than those with a weak health risk perception. The results indicate that the high-aged with strong health risk perception have stronger motivation to purchase blockchain traceable fresh fruits; that is, health risk perception has a greater impact on the high-aged to buy blockchain traceable fresh fruits. There are two possible reasons. One is that the young perceive a lower health risk than the older [40], and the other might be that the young rated the fruit a lower health risk perception than the older [40], so they pay more attention to the food-related to health issues and prefer buying more safe food.

From the income grouping, health risk perception is significantly positive for the intention of the low-income and high-income consumers to purchase blockchain traceable fresh fruits at the 5% and 1% level, respectively. The marginal effect value shows that low-income consumers with strong health risk perception have a 5.3% higher likelihood of purchasing blockchain traceable fresh fruits than those with a weak health risk perception. In comparison, high-income consumers with strong health risk perception have a 6.9% higher likelihood of purchasing blockchain traceable fresh fruits than those with a weak health risk perception. This shows that the high-income consumers with strong health risk perception have stronger motivation to purchase blockchain traceable fresh fruits; that is, health risk perception has more influence on the high-income consumers to purchase blockchain traceable fresh fruits. It implies that people with higher income pay more attention to food quality and safety and can pay the premium price to ensure food quality-related concerns to consume products with more health attributes. On the contrary, lower-income people engage in more poor health behaviors [68]. Furthermore, health risk perception differs by household income level, and income is a significant positive predictor of health risk perception [68]. These findings imply that traceable food products might experience exponential expansion and acceptance in China as the middle-income group has acquired a more stable income.

Among the education grouping, health risk perception is significantly positive at the statistical levels of 10% and 1% for the low-educated and high-educated consumers to purchase traceable fresh fruits, respectively. From the perspective of the marginal effect, the low-educated consumers with strong health risk perception have a 5.1% greater likelihood of purchasing blockchain traceable fresh fruits than those with a weak health risk perception. Likewise, the high-educated consumers with strong health risk perception have a 6.3% higher likelihood of purchasing blockchain traceable fresh fruits than those with a weak health risk perception. This suggests that highly educated consumers have strong health risk perception and show stronger motivation to purchase blockchain traceable fresh fruits; that is, health risk perception has a greater impact on highly educated consumers to purchase blockchain traceable fresh fruits. The possible reason is that education and knowledge prove to be an adequate tool for health risk perception, making the consumers adopt safe food [58]. Previous studies also noted that a higher education level is associated with greater risk perception [69].

### 4.3. Robustness Test

The robustness of the empirical results was examined using three methods of step-wise robustness checks. First, we applied the Oprobit model for estimation. Second, we remeasured consumers’ health risk perception by including the following statement: “at present, fresh fruits containing chemicals account for a large part of the fruit market due to industrial and/or waste and/or water pollution”. Responses were noted on a 5-point Likert-type scale ranging from 1 (extremely disagree) to 5 (extremely agree). We then carried out the regression with the Ologit model and the Oprobit model, respectively. Lastly, we changed the purchase intention of the dependent variable from an ordered five-category variable to an ordered three-category variable; that is, the “extremely unlikely” and “unlikely” in the responses are defined as “unlikely”, “likely”, and “extremely likely” are defined as “likely”, which are assigned 1 and 3, respectively, and the “uncertain” response is still assigned 2. The Ologit model and the Oprobit model were then used for estimation, respectively. The robustness test results are shown in Table 4. The health risk perception variable passed the significance test, and the coefficient was positive, which supports the conclusion that health risk perception has a significant positive impact on consumers’ purchase intention of blockchain traceable fresh fruits.

## 5. Conclusions

The fresh fruit traceability system based on blockchain technology helps overcome the information asymmetry between the food suppliers and consumers and provides sufficient information to facilitate safe food purchase decisions. Notably, due to multiple factors, the development of blockchain traceability lags behind the technical level in terms of promotion practice. Based on the online-survey data of consumers in the traceability system pilot cities, we empirically tested the impact of health risk perception on purchase intention with regard to blockchain traceable fresh fruits. The results show that health risk perception significantly affects consumers’ purchase intentions of blockchain traceable fresh fruits. It indicates that the stronger the consumers’ health risk perception, the stronger their purchase intention for traceable fresh fruits. The impact of health risk perception on the purchase intention of blockchain traceable fresh fruits is heterogeneous in gender, age, income and education, which is also related to its advanced technical attributes to a certain extent, and can also explain the lag of blockchain traceability application. The impact of males’ health risk perception on purchase intention is stronger than females, and the impact of health risk perception on the purchase intention of older consumers is stronger than that of younger ones. The health risk perception of high-income consumers had a stronger impact on purchase intention than low-income consumers, and the health risk perception of highly educated consumers had a stronger impact on purchase intention than on lesser-educated ones. Family member structure, consumers’ traceability cognition and purchase experience of traceable products are also among the important factors affecting consumers’ purchase intention with regard to blockchain traceable fresh fruits in China.

## 6. Implications

Based on the above findings, the following policy implications can be drawn. The application of blockchain in food supply chain traceability has broad prospects, but there are still few projects involving blockchain in the field of food traceability, and most of the projects have been implemented as pilot projects [26,27]. Therefore, the development, acceptance, and application of blockchain traceability systems should be accelerated at the provincial and national levels in China, especially for the high-educated and high-income groups. Furthermore, at the moment, food traceability covers a few areas and products which need to expand as it holds sufficient potential for changing marketing systems and their transformation. Continued expansion and coverage of traceable products are recommended by giving a full play to the function of the traceability system to reveal product quality and safety information, reduce consumers’ health risk perception and enhance consumers’ trust in product quality and safety. From the perspective of stakeholders advocating the implementation of traceability systems, producers should be actively encouraged to adopt blockchain technology for product traceability to improve product circulation efficiency and market competitiveness. Blockchain traceability systems can even be combined with other supply chain management systems for product sales forecasting or order planning [70]. Likewise, there is a dire need to strengthen the traceability system and its promotion in order to expand its acceptance to improve consumers’ cognition and trust in blockchain traceable products. In addition, different marketing strategies should be implemented for different target groups during promotions. The study findings suggest that a wider application of the food traceability system would benefit all of the stakeholders along the supply chain—farmers, traders, processors, and consumers—by ensuring improved incomes for suppliers and safe and trusted food products on the consumer end. Thus, at the earlier stage of food traceability development, targeting high-income cities and societies with a higher educational proportion might help realize rapid awareness, acceptance, and promotion and set it as a benchmark for other Chinese cities.

There are only a few blockchain traceable products in the current market; therefore, we cannot understand the real purchase behavior of consumers, and the purchase intention does not necessarily lead to purchase behavior. There is a gap between these two. Furthermore, studies are needed to explore consumers’ real purchase behavior of blockchain traceable fresh fruits through real auction experiments, which might provide more practical insights for policy and practice.

## Figures and Tables

**Table 1:** Variable definition and descriptive statistics.

Variables	Definition	Mean	SD	Min	Max
**Dependent variable**					
Purchase intention	Possibility of purchasing blockchain traceable fresh fruit, assign from 1 to 5 in turn	4.174	0.745	1	5
**Independent variable of interest**					
Health risk perception	Value of attitude towards fresh fruit quality and safety, assign from 1 to 5 in turn	4.093	0.793	1	5
**Control variables**					
** Individual characteristics**					
Gender	Female = 1, male = 0	0.604	0.489	0	1
Age	the high school and below educated = 1, the college educated = 2, the graduate educated or above = 3	1.560	0.559	1	3
Education	young (29 years and under) = 1, the middle-aged (30~49 years old) = 2, the elderly (50 years and above) = 3	2.028	0.414	1	3
Occupation	Whether occupation related to food industry, yes = 1, no = 0	0.100	0.300	0	1
Marriage	Married = 1, unmarried = 0	0.422	0.494	0	1
** Family characteristics**					
Family scale	Total household population/person	3.955	1.400	1	20
Number of children	family with fewer children (with 1 children and under) = 1, family with two children = 2, family with more children (with 3 children and above) = 3	1.757	0.662	1	3
Family income	low-income (less than $7450/year) = 1, middle income ($7.450~22,350/year) = 2, high-income (more than $22,350/year) = 3	2.411	0.667	1	3
** Individual cognitive experience**					
Traceability cognition	Whether heard of food traceability system of traceable food, yes = 1, no = 0	0.847	0.360	0	1
Purchase experience	Whether purchased traceable fresh agricultural products, yes = 1, no = 0	0.832	0.374	0	1
Food poisoning experience	Whether experienced food poisoning or not. Yes = 1, no = 0	0.063	0.244	0	1

Note: With references to existing studies [58,59], 5-point Likert-type scale was introduced to measure related variables.

**Table 2:** Impact of health risk perception on purchase intention.

Variables	OLS Model			Ologit Model (Marginal Effects)		
	(1)	(2)	(3)	(4)	(5)	(6)
**Independent variable of interest**						
Health risk perception	0.153 *** (0.030)	0.108 *** (0.029)	0.108 *** (0.029)	0.092 *** (0.018)	0.064 *** (0.017)	0.064 *** (0.017)
**Control variables**						
Gender		−0.040 (0.044)	−0.038 (0.044)		−0.027 (0.026)	−0.026 (0.026)
Age (Elderly is the reference group)						
Young		0.040 (0.133)	0.021 (0.134)		0.016 (0.080)	0.005 (0.081)
Middle-aged		−0.022 (0.134)	−0.035 (0.134)		−0.023 (0.080)	−0.032 (0.081)
Education (The high school and below educated is the reference group)						
College-educated		0.209 ** (0.106)	0.215 ** (0.105)		0.122 ** (0.051)	0.125 ** (0.050)
Graduate educated or above		0.294 ** (0.125)	0.314 ** (0.127)		0.192 *** (0.065)	0.206 *** (0.064)
Occupation		−0.129 (0.083)	−0.135 (0.082)		−0.054 (0.045)	−0.057 (0.045)
Marriage		−0.068 (0.047)	−0.067 (0.047)		−0.037 (0.027)	−0.037 (0.027)
Family scale		−0.022 (0.018)	−0.025 (0.018)		−0.012 (0.011)	−0.014 (0.011)
Number of children (family with fewer children is the reference group)						
With two children		0.157 *** (0.058)	0.154 *** (0.058)		0.088 *** (0.033)	0.086 *** (0.033)
With more children		0.042 (0.091)	0.029 (0.091)		0.034 (0.051)	0.028 (0.051)
Family income (low-income is the reference group)						
Middle-income		0.201 ** (0.091)	0.199 ** (0.091)		0.105 ** (0.047)	0.104 ** (0.047)
High-income		0.191 ** (0.091)	0.192 ** (0.091)		0.099 ** (0.047)	0.101 ** (0.047)
Traceability cognition		0.272 *** (0.067)	0.273 *** (0.068)		0.160 *** (0.037)	0.161 *** (0.037)
Purchase experience		0.321 *** (0.063)	0.319 *** (0.063)		0.194 *** (0.034)	0.191 *** (0.035)
Food poisoning experience		−0.006 (0.092)	−0.014 (0.092)		0.005 (0.052)	0.001 (0.052)
_cons	3.546 *** (0.127)	2.921 *** (0.214)	3.012 *** (0.221)	—	—	—
Regions effect	Uncontrolled	Uncontrolled	Controlled	Uncontrolled	Uncontrolled	Controlled
R^2^	0.027	0.137	0.139	—	—	—
Pseudo R^2^	—	—	—	0.013	0.067	0.068
Wald chi	—	—	—	25.17	124.52	130.49
Number of obs	1058	1058	1058	1058	1058	1058

Notes: The values in parentheses are robust standard errors; *** and ** represent 1% and 5% statistical significance levels, respectively.

**Table 3:** Results of Heterogeneity analysis.

Variables	Grouped by Gender (Marginal Effect)		Grouped by Age (Marginal Effect)		Grouped by Income (Marginal Effect)		Grouped by Education (Marginal Effect)	
	Male	Female	Low-Aged	High-Aged	Low-Income	High-Income	Low-Educated	High-Educated
**Independent variable of interest**								
Health risk perception	0.101 *** (0.026)	0.045 ** (0.022)	0.048 ** (0.022)	0.080 *** (0.026)	0.053 ** (0.026)	0.069 *** (0.024)	0.051 * (0.030)	0.063 *** (0.020)
**Control variables**	Controlled	Controlled	Controlled	Controlled	Controlled	Controlled	Controlled	Controlled
Regions effect	Controlled	Controlled	Controlled	Controlled	Controlled	Controlled	Controlled	Controlled
Pseudo R^2^	0.076	0.079	0.092	0.061	0.057	0.096	0.078	0.070
Wald chi	63.76	94.14	106.05	54.62	58.85	116.19	48.29	109.99
Number of obs	419	639	556	502	516	542	267	791

Notes: The values in parentheses are robust standard errors; ***, ** and * represent 1%, 5% and 10% statistical significance levels, respectively.

**Table 4:** Results of robustness test.

Variables	Replace Model	Replace Independent Variable of Interest		Replace Dependent Variable	
	Oprobit Model	Ologit Model	Oprobit Model	Ologit Model	Oprobit Model
**Independent variable of interest**					
Health risk perception	0.060 *** (0.016)	0.025 * (0.013)	0.025 * (0.013)	0.047 *** (0.013)	0.046 *** (0.012)
**Control variables**	Controlled	Controlled	Controlled	Controlled	Controlled
Regions effect	Controlled	Controlled	Controlled	Controlled	Controlled
Pseudo R^2^	0.067	0.063	0.062	0.118	0.117
Wald chi	132.99	121.59	123.04	111.00	120.14
Number of obs	1058	1058	1058	1058	1058

Notes: The values in parentheses are robust standard errors; *** and * represent 1% and 10% statistical significance levels, respectively.

## Data Availability

Data available from the authors upon request.
